# Preface

**DOI:** 10.1093/jac/dkab197

**Published:** 2021-08-01

**Authors:** Alan Johnson, Lewis Russell, John D Perry, Monica Slavin, Evelina Tacconelli

It is now widely recognized that inappropriate use of antibiotics is a major driver of the emergence and spread of antibiotic resistance. This has resulted in the development of antibiotic stewardship programmes, which aim to optimize antibiotic use by maximizing the clinical benefits while minimizing adverse consequences including toxicity and resistance. As antibiotic resistance is a global threat, the formulation of stewardship programmes and assessment of their cost-effectiveness requires knowledge of the trends in antibiotic usage at local, national and international levels. For many years, a major contribution to the surveillance of antibiotic use has been provided by the European Surveillance of Antimicrobial Consumption Network (ESAC-Net, formerly ESAC), which comprises an international network of surveillance systems that collects data on antibiotic consumption across the EU/European Economic Area (EEA). This Supplement comprises a series of articles presenting data on antibiotic consumption in the community from 30 EU/EEA countries over two decades (1997 to 2017), and updates previously available information covering the periods 1997–2003 and 1997–2009. The articles review temporal trends, seasonal variation, presence of change-points and changes in the composition of the main antibiotic groups. Data outputs are presented as yearly antibiotic consumption aggregated at the level of the active substance, using the WHO ATC classification and expressed in DDD (ATC/DDD index 2019) per 1000 inhabitants per day, analysed using a range of non-linear mixed models. For those readers not fully conversant with such statistical methodology, the Supplement also includes a tutorial that describes and illustrates statistical methods for analysing time trends (including abrupt changes, referred to as change-points) in antibiotic consumption. These updated analyses of two decades of ESAC-Net data provide the most comprehensive and detailed description yet of antibiotic consumption in the community in Europe, which we hope will be of interest to JAC readers, and in particular those involved in informing and evaluating control strategies.

## Transparency declarations

None to declare.

